# Combining Novel Hormonal Therapies with a Poly (ADP-Ribose) Polymerase Inhibitor for Metastatic Castration-Resistant Prostate Cancer: Emerging Evidence

**DOI:** 10.3390/curroncol30120751

**Published:** 2023-12-04

**Authors:** Jie Yang, Xingyu Xiong, Weitao Zheng, Xinyang Liao, Hang Xu, Lu Yang, Qiang Wei

**Affiliations:** Department of Urology, Institute of Urology, Center of Biomedical Big Data and National Clinical Research Center for Geriatrics, West China Hospital of Sichuan University, Chengdu 610041, China

**Keywords:** poly (ADP-ribose) polymerase inhibitor, mCRPC, first-line therapy, homologous recombination repair

## Abstract

Preclinical and clinical studies have suggested potential synergies of combining poly (ADP-ribose) polymerase (PARP) inhibitors and novel hormonal therapies (NHT) for patients with metastatic castration-resistant prostate cancer (mCRPC). We systematically searched PubMed, ClinicalTrials.gov and ASCO-GU annual meeting abstracts up to March 2023 to identify potential phase III trials reporting the use of combining PARP inhibitors with NHT in the first-line setting for mCRPC. A total of four phase III trials met the criteria for subsequent review. Emerging data suggested that the radiographic progression-free survival (rPFS) was significantly longer in the PARP inhibitor combined with NHT group versus the placebo plus NHT group for the first-line setting of biomarker-unselected mCRPC patients, especially for patients with homologous recombination repair (HRR) mutation (HRR m), and with the greatest benefit for BRCA1/2 mutation (BRCA1/2 m) populations. Final overall survival (OS) data of the PROpel trial indicated a significant improvement in median OS for mCRPC patients with HRR m and BRCA1/2 m receiving olaparib + abiraterone. Prior taxane-based chemotherapy might not influence the efficacy of the combination. Compared with the current standard-of-care therapies, combining NHT with PARP inhibitors could achieve a significant survival benefit in the first-line setting for mCRPC patients with HRR and BRCA1/2 mutations.

## 1. Introduction

Metastatic castration-resistant prostate cancer (mCRPC) is complicated and lethal. Androgen deprivation therapy (ADT), suppressing the secretion of testicular androgens in different ways, is the cornerstone of treatment for metastatic prostate cancer. Despite great benefits and improvements in patients’ survival outcomes, once on ADT the development of mCRPC is just a matter of time and an adaptive mechanism for tumor cells to maintain high intracellular androgen level and overexpress androgen receptor (AR) has been observed in mCRPC [1]. Under this circumstance, new androgen receptor pathway inhibitors targeting the androgen axis called novel hormonal therapies (NHT) have been proved to be promising agents in the combination with ADT. For example, abiraterone acetate plus prednisone (AAP) and enzalutamide (ENZA) have been approved in the first-line therapy setting of mCRPC according to the European Association of Urology (EAU) guidelines [2]. However, after a period of ADT + NHT treatment, almost all patients will develop drug resistance, and NHT-resistant mCRPC is featured with a high malignant degree, a lack of treatment modality and a poor prognosis [3]. In clinical trial settings, overall survival (OS) is about 3 years in patients with mCRPC receiving the current first-line therapies recommended by the EAU and National Comprehensive Cancer Network (NCCN) prostate cancer guidelines [2,4,5,6,7]. OS is even shorter for these patients in a real-world setting, and nearly 50% of patients receive only one-line life-prolonging therapy [8]. There is an emerging need for new treatment modalities with longer response durations in the first-line therapy setting for patients with mCRPC.

Approximately 30% of patients with mCRPC harbor somatic and/or germline alterations in homologous recombination repair (HRR)-associated genes [9]. Previous phase II and III trials have demonstrated the efficacy of poly (ADP-ribose) polymerase (PARP) inhibitors in mCRPC with the HRR mutation (HRR m), especially for patients with deleterious BRCA1/2 mutation (BRCA1/2 m), as a second- or third-line therapy [10,11,12,13,14,15,16]. Based on these trials, two PARP inhibitors, olaparib and rucaparib, have been approved in the US and EU for patients with a deleterious or suspected deleterious HRR m or BRCA1/2 m who progressed following prior NHT and/or taxane-based chemotherapy [17,18,19].

Preclinical evidence has suggested crosstalk between AR and PARP pathways [20,21,22]. PARP inhibitors could influence transcriptional changes induced by AR pathways and increase the sensitivity of NHT [20]. Similarly, NHT could inhibit the transcription of some HRR genes and increase the treatment efficacy of PARP inhibitors [21,22]. A phase II trial confirmed these preclinical findings [23], which included patients with mCRPC who progressed following prior docetaxel treatment, and found that olaparib in combination with AAP significantly achieved a longer investigator-assessed radiographic progression-free survival (rPFS) than AAP alone in patients with or without HRR m. Therefore, several randomized phase III trials were conducted to test the combination of PARP inhibitors and NHT in patients with treatment-naive mCRPC who were unselected by HRR mutation status.

The aim of this systematic review is to summarize the emerging evidence of the combination of PARP inhibitors and NHT as a first-line therapy in biomarker-unselected patients with mCRPC based on current phase III trials. Based on current data, we will try to preliminarily answer the following questions: Should the combination now become standard-of-care for first-line mCRPC? Could previous NHT and taxane-based chemotherapy at the metastatic castration-sensitive prostate cancer (mCSPC) and/or non-metastatic castration-resistant prostate cancer (nmCRPC) stage influence the efficacy of the combination?

## 2. Methods

We conducted the current systematic review following the Preferred Reporting Items for Systematic Reviews and Meta-analyses (PRISMA) reporting guideline [24] and the methodology of the European Association of Urology (EAU) for conducting a systematic review [25]. The study was not registered in a database such as PROSPERO.

We systematically searched PubMed, ClinicalTrials.gov and American Society of Clinical Oncology Genitourinary Cancers Symposium (ASCO-GU) annual meeting abstracts up to March 2023. The following searching terms were used: [“Prostate Cancer”] AND [“PARP inhibitor” OR “Poly (ADP-Ribose) Polymerase” OR “Olaparib” OR “Rucaparib” OR “Talazoparib” OR “Niraparib” OR “Veliparib”]. We performed the study eligibility for published articles and ASCO abstracts using the population, intervention, comparator, outcome and study (PICOS) approach: (P) studies focused on patients with biomarker-unselected mCRPC; (I) who received PARP inhibitors combined with NHT as a first-line therapy for mCRPC; (C) in which NHT was used as a comparator; (O) reporting oncologic outcomes and/or adverse effects (AEs); (S) in phase III trials. Additionally, relevant on-going phase III trials, which have not reported relevant outcomes, were also included.

Two reviewers independently screened all of our searching results to include studies. For trials with relevant outcomes, two reviewers independently extracted the following data from the included trials: author, the NCT number, year of publication, sample size, baseline patients and tumor characteristics, HRR m status, treatment modalities at the mCSPC and/or nmCRPC stage, type and dose of PARP inhibitors, type of combined NHT, relevant oncologic outcomes and AEs. For on-going trials which have not reported relevant data, we would summarize their protocols. Any disagreements were resolved by a third reviewer.

## 3. Results

### 3.1. Study Selection

**Figure 1:**
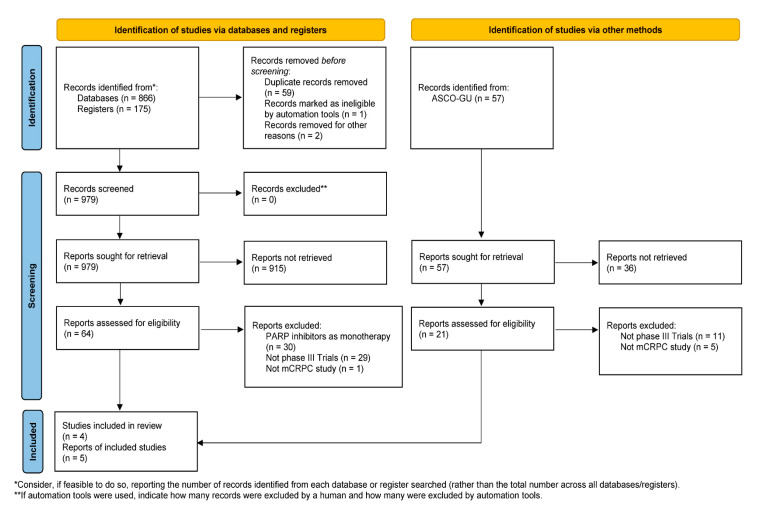
Preferred Reporting Items for Systematic Reviews and Meta-analysis flow diagram of the study selection. PARP = poly (ADP-ribose) polymerase; mCRPC = metastatic castration-resistant prostate cancer; ASCO-GU = American Society of Clinical Oncology Genitourinary Cancers Symposium. From Ref. [24]. For more information, visit: http://www.prisma-statement.org/ (accessed on 18 October 2023).

A total of 866 publications, 175 on-going clinical trials and 57 ASCO-GU abstracts were identified for eligibility. After assessment by title and abstract, 85 were included for subsequent review, of which 76 were excluded as they were not phase III trials, did not include mCRPC patients, and used PARP inhibitors as monotherapy. Three publications [26,27,28], one on-going clinical trial [29] and five ASCO-GU abstracts [30,31,32,33,34] were included in the current systematic review [Figure 1] [Supplemental Table S1].

### 3.2. Characteristics of Included Trials

Four phase III trials were conducted to assess the role of combining PARP inhibitors and NHT as a first-line therapy for biomarker-unselected mCPRC patients and were included for further evaluation. The details of the baseline characteristics of the patients in the four trials are displayed in Table 1.

The MAGNITUDE study [26,30,31] included treatment-naive mCRPC patients to test the combination of niraparib and abiraterone. In the MAGNITUDE trial, patients received 1000 mg abiraterone plus 200 mg niraparib or 1000 mg abiraterone plus placebo once daily. Notably, patients were firstly divided into HRR m and non-HRR mutation (non-HRR m) cohort, and then were randomly assigned in a 1:1 ratio to receive the combination therapy or abiraterone alone. There were 31 (3.1%) and 85 (20.1%) patients who received NHT and taxane chemotherapy for mCSPC or nmCRPC, respectively. The primary end point was centrally reviewed radiographic progression-free survival (rPFS). It should be noted that the MAGNITUDE trial allowed up to 4 months of AAP for first-line mCRPC before random assignment [Table 1]. 

The PROpel study [27,32] tested the combination of abiraterone (1000 mg once daily) and olaparib (300 mg twice daily) in biomarker-unselected treatment-naive mCRPC patients. HRR mutation status was tested after randomization. Only one patient received NHT for mCSPC. There were 90 (22.6%) and 89 (22.4%) patients who received docetaxel for mCSPC in the combination arm and placebo arm, respectively. The primary end point was investigator-assessed rPFS [Table 1]. 

The TALAPRO-2 study [28,33] also included biomarker-unselected treatment-naive patients with mCRPC. In the TALAPRO-2 trial, patients received 0.5 mg talazoparib plus 160 mg enzalutamide (ENZA) or placebo plus 160 mg ENZA once daily. Blood samples or the most recent tumor tissue samples for the HRR assessment were collected before randomization. Twenty-one (5.2%) and twenty-five (6.2%) patients received AAP at the mCSPC stage in the combination arm and the ENZA arm, respectively. As for docetaxel chemotherapy at the mCSPC stage, there were 86 (21.4%) and 93 (23.1%) patients in each arm, respectively. The primary end point was also investigator-assessed rPFS [Table 1].

The last trial was the CASPAR study [29,34], which has not reported any results. The CASPAR trial was designed to test the combination of ENZA (160 mg once daily) and rucaparib (600 mg twice daily) in biomarker-unselected treatment-naive mCRPC patients. Prior NHT (except ENZA) and/or docetaxel chemotherapy at the mCSPC and/or nmCRPC stage was allowed. The primary end points were rPFS and OS [Table 1]. 

### 3.3. Evidence for Combining PARP Inhibitors and NHT as First-Line Therapy for mCRPC 

#### 3.3.1. rPFS

**Figure 2:**
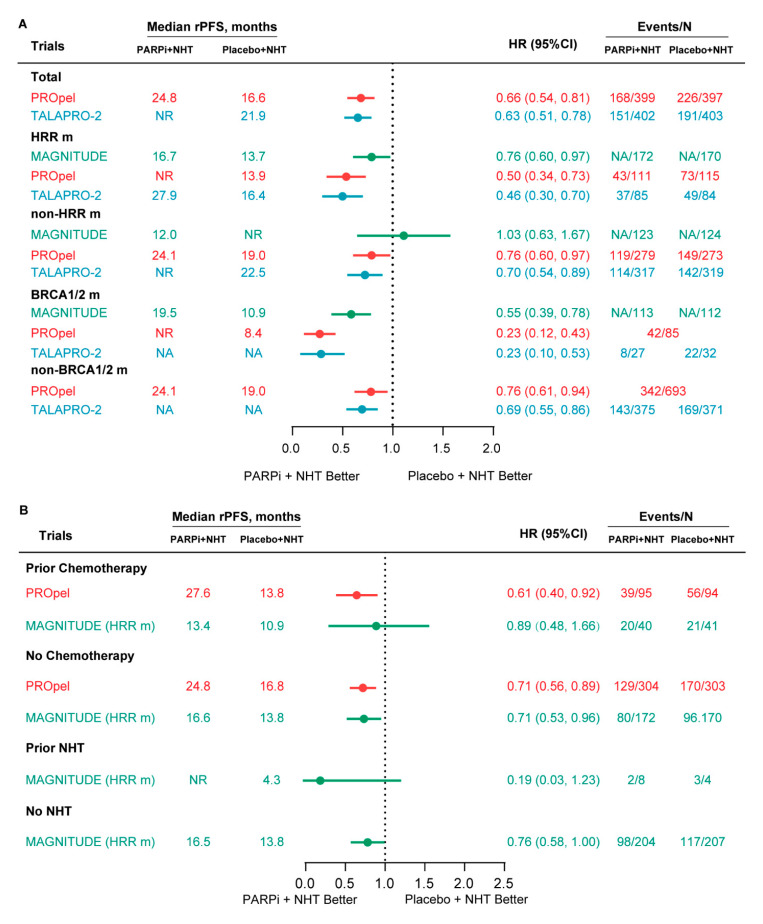
Forest plot of rPFS for patients receiving the combination and standard-of-care therapies. (**A**) rPFS for biomarker-unselected and biomarker-selected patients; (**B**) rPFS for subgroup stratified by prior NHT and taxane-based chemotherapy in the mCSPC and/or nmCRPC setting. rPFS = radiographic progression-free survival; NR = not reached; NHT = novel hormonal therapies; PARPi = poly (ADP-ribose) polymerase inhibitors; HRR = homologous recombination repair; HRR m = HRR mutation; BRCA1/2 m = BRCA1/2 mutation; N = number; NA = no data; HR = hazard ratio.

For biomarker-unselected treatment-naive mCRPC patients, both PROpel and TALAPRO-2 trials showed significantly longer median rPFS in the combination arm than in the placebo arm (olaparib + AAP vs. placebo + AAP: 24.8 vs. 16.6 months; hazard ratio (HR), 0.66; 95% CI, 0.54 to 0.81. Talazoparib + ENZA vs. placebo + ENZA: not reached (NR) vs. 21.9 months; HR, 0.63; 95% CI, 0.51 to 0.78) [Figure 2A]. All HR for HRR m and non-HRR m populations significantly favored the combination arm in the PROpel and TALAPRO-2 trials [Figure 2A]. In the PROpel trial, patients were also stratified into BRCA1/2 m and non-BRCA1/2 mutation (non-BRCA1/2 m) subgroups, and subgroup analyses also indicated that the olaparib + AAP achieved a significantly longer rPFS than placebo + AAP regardless of the status of BRAC1/2 [Figure 2A]. In the MAGNITUDE trial, niraparib + AAP could only achieve a significantly longer median rPFS in HRR m and BRCA1/2 m populations [Figure 2A]. As the MAGNITUDE trial allowed patients to receive up to 4 months of prior AAP for mCRPC, sensitivity analyses showed consistent results in both HRR m and BRCA1/2 m populations after excluding these patients [Supplemental Table S2]. In the placebo + NHT arm, the median rPFS was shorter in the HRR m and BRCA1/2 m subgroup than that in the non-HRR m and non-BRCA1/2 m subgroup among the three trials [Figure 2A]. Interestingly, the combination arm achieved inverse results among the four subgroups [Figure 2A].

In the PROpel trial, patients were also stratified by whether they received taxane-based chemotherapy at the mCSPC stage. For subgroups with or without prior chemotherapy, the median rPFS was significantly longer in the combination arm than that in the placebo + AAP arm [Figure 2B]. The MAGNITUDE trial divided patients of the HRR m cohort into four subgroups which were with or without prior taxane-based chemotherapy and with or without prior NHT in the mCSPC setting. Subgroup analyses indicated that niraparib + AAP could only achieve a significantly longer median rPFS than placebo + AAP in patients with HRR m who had not been treated with taxane-based chemotherapy and NHT at the mCSPC stage [Figure 2B].

#### 3.3.2. OS

**Figure 3:**
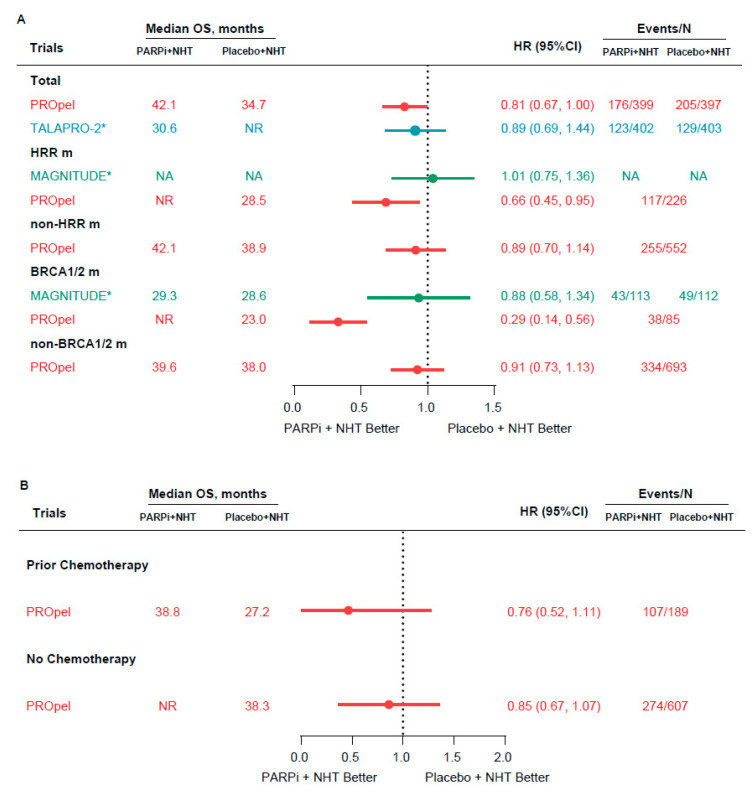
Forest plot of OS for patients receiving the combination and standard-of-care therapies. (**A**) OS for biomarker-unselected and biomarker-selected patients; (**B**) OS for subgroup stratified by prior taxane-based chemotherapy in the mCSPC setting. OS = overall survival; NR = not reached; NHT = novel hormonal therapies; PARPi = poly (ADP-Ribose) polymerase inhibitors; HRR = homologous recombination repair; HRR m = HRR mutation; BRCA1/2 m = BRCA1/2 mutation; N = number; NA = no data; HR = hazard ratio. * Immature data.

Only the PROpel trial reported the final OS data of included patients. For biomarker-unselected treatment-naive mCRPC patients, there was a trend that olaparib + AAP could achieve an OS benefit compared with placebo + AAP (median OS, 42.1 vs. 34.7 months; HR, 0.81; 95% CI, 0.67 to 1.00) [Figure 3A]. Compared with the placebo + AAP arm, HR for HRR m and BRCA1/2 m populations significantly favored the combination arm, especially for the BRCA1/2 m populations (median OS, NR vs. 23.0 months; HR, 0.29; 95% CI, 0.14 to 0.56) [Figure 3A]. As for non-HRR m and non-BRCA1/2 m populations, olaparib + AAP only showed a benefit of 3.2 and 1.6 months in median OS compared with placebo + AAP [Figure 3A]. Similarly, the median OS was also shorter in the HRR m and BRCA1/2 m subgroups than in the non-HRR m and non-BRCA1/2 m subgroups in the PROpel trial, when patients were treated with placebo + AAP. Additionally, the results were also inverse in the combination arm [Figure 3A]. The immature OS data from the TALAPRO-2 trial also favored the talazoparib + ENZA among biomarker-unselected mCRPC patients in the first-line setting (HR, 0.89; 95% CI, 0.69 to 1.44) [Figure 3A]. As for the MAGNITUDE trial, there was only a trend towards improved OS with niraparib + AAP in the BRCA1/2 m populations based on the immature OS data [Figure 3A]. When stratified by prior taxane-based chemotherapy in the mCSPC setting, the PROpel trial showed consistent trends towards OS benefit in the combination arm [Figure 3B].

#### 3.3.3. Safety

The incidence of grade ≥ 3 AEs was 66.9%, 47.2% and 75.2% with combination therapy in the MAGNITUDE, PROpel and TALAPRO-2 trial, respectively [Table 2]. In the placebo + NHT arm, the incidence of grade ≥ 3 AEs in the three trials was 46.5%, 38.4% and 45.1%, respectively. The most common grade 3 or higher AEs was anemia (MAGNITUDE: 29.7% vs. 7.6%; PROpel: 15.1% vs. 3.3%; TALAPRO-2: 46.5% vs. not reported) in the combination arm versus the placebo arm [Table 2]. It should be noted that grade 3 or higher AEs in the blood and lymphatic system were more common in the combination arm among the three trials [Table 2]. AEs leading to interruption, dose reduction and discontinuation are also displayed in Table 2.

## 4. Discussion

Based on current data from three phase III trials, rPFS was significantly longer in the PARP inhibitor combined with NHT group versus the placebo plus NHT group for the first-line setting of biomarker-unselected mCRPC patients. The combination could achieve a greater benefit in HRR m populations and with the greatest benefit in the BRCA1/2 m populations. As for OS, the final OS data of PROpel indicated a significant benefit of the combination in HRR m and BRCA1/2 m populations. Immature OS of the MAGNITUDE and TALAPRO-2 trials also showed a trend towards improved OS in HRR m and biomarker-unselected mCPRC patients who received PARP inhibitors combined with NHT in the first-line setting, respectively. Additionally, current data suggested that prior taxane-based chemotherapy at the mCSPC stage might not influence the efficacy of the combination in the first-line setting of mCRPC.

The current results were also consistent with a previous phase II trial which evaluated the efficacy of abiraterone plus olaparib versus abiraterone plus placebo in biomarker-unselected mCPRC patients who had previously received docetaxel (HR, 0.65; 95% CI, 0.44 to 0.97) [23]. Moreover, the role of AAP and ENZA in the first-line setting for biomarker-unselected mCPRC patients was also expected. The median rPFS of AAP and ENZA was 16.5 and 20 months for mCRPC patients without chemotherapy in COU-AA-302 and PREVAIL, respectively [4,35], which was also consistent with the 16.6 and 21.9 months reported in PROpel and TALAPRO-2. These results further suggested that AAP + olaparib and ENZA + talazoparib could significantly improve rPFS beyond the current standard first-line therapy in biomarker-unselected mCPRC patients.

Nevertheless, the MAGNITUDE trial showed that niraparib + AAP could only extend rPFS benefit in HRR m and BRCA1/2 m populations. The following reasons might partially explain the conflicting results between MAGNITUDE and PROpel trials in non-HRR m populations: First, the dose of olaparib in PROpel was the same as that which was used in monotherapy studies (300 mg twice daily) [11,12]. The dose of niraparib in MAGNITUDE was 200 mg once daily while it was used 300 mg once daily in monotherapy studies [16]. The dose of niraparib in MAGNITUDE was based on the results of the phase Ib BEDIVERE trial, and the 200 mg niraparib in combination with AAP was selected after considering the pharmacokinetic results and safety profile [36]. The dose reduction might influence the potency of niraparib for inhibiting PARP1. Second, the PARP inhibitors used in the two trials were also different. The potential synergies might be different when combining AAP with different types of PARP inhibitors. Third, in the MAGNITUDE trial, nearly one in six patients in the non-HRR m cohort were allowed to receive up to 4 months of prior AAP for mCRPC. According to our sensitivity analysis for HRR m cohort, patients receiving AAP over 2 months for mCRPC might limit the efficacy of niraparib + AAP compared with placebo + AAP.

All of the three trials indicated that the median rPFS in HRR m and BCRA1/2 m populations who received NHT alone was shorter than in non-HRR m and non-BRCA1/2 m populations. These results were also consistent with prior studies which reported that standard therapy had poor prognosis and worse treatment outcomes in patients with BRCA1/2 m [37,38]. Notably, the combination therapy achieved a longer median rPFS in HRR m and BCRA1/2 m populations than in non-HRR m and non-BRCA1/2 m populations. These results indicated that patients with HRR m might benefit more from receiving PARP inhibitors combined with NHT as first-line therapy in the mCRPC setting.

The final OS data of PROpel suggested that combining olaparib with AAP could achieve a 42.1 months median OS in biomarker-unselected mCRPC patients, which was the longest median OS reported to date in the phase III trials for first-line therapy of mCRPC. Additionally, the median OS of placebo + AAP in PROpel was 34.7 months, which was the same as in COU-AA-302 [4]. Trends towards OS benefit of olaparib + AAP were observed in both biomarker-unselected patients and HRR m, non-HRR m, BRCA m and non-BRCA m subgroups. Consistent with the rPFS data, the median OS of AAP in HRR m and BRCA1/2 m populations was shorter than in non-HRR m and non-BRCA1/2 m populations, and olaparib + AAP could achieve a longer median OS in the mutation populations. It should be noted that, compared with AAP, olaparib + AAP could only significantly improve OS in HRR m and BRCA1/2 m populations, and the greatest benefit was achieved in BRCA1/2 m populations. Additionally, immature OS data of TALAPRO-2 and MAGNITUDE also illustrated trends to favor the combination in biomarker-unselected and BRCA1/2 m patients, respectively.

Not surprisingly, there were more grade ≥ 3 AEs in the combination arm compared with the placebo arm among the three trials, especially in TALAPRO-2 (75.2% grade ≥ 3 AEs in talazoparib + ENZA arm). Grade ≥ 3 anemia was the most common AEs, and there were 46.5% grade ≥ 3 anemia happening in patients treated with talazoparib + ENZA. However, AEs profiles for the combination therapy were consistent with their known individual toxicity profiles and did not suggest new safety signals that affected the benefit–risk profile.

However, a significant question which could limit the application in real clinical practice was the paucity of the patients who received AR-target agents in the mCSPC and/or nmCRPC setting (this constituted only 3.1%, 0.3% and 5.2% of the MAGNITUDE, PROpel and TALAPRO-2 populations, respectively). Given the new standard use of novel AR pathway inhibitors in the management of mCSPC in current practice [39,40], the results from these trials could not be broadly applied to patients reaching the first-line mCRPC state who received prior ADT combined with NHT (including enzalutamide/apalutamide/darolutamide) for mCSPC. Likewise, due to the exclusion of patients who previously received abiraterone at the mCSPC stage in the MAGNITUDE and PROpel trials, the conclusions were not powerful enough to expand to the real-world medical setting as well [41]. On the basis of the data from phase III trials, we can propose that germline and somatic testing in all individuals with metastatic prostate cancer is necessary to maximize the positive effects of the combination therapy of PARP inhibitors plus NHT, especially for those who harbor HRR gene alteration and BRCA1/2 mutation.

PARP inhibitors combined with NHT present a representative example of combination therapy, which is a promising approach to overcome drug resistance in the management of prostate cancer at an advanced stage [42]. Increasing evidence has emerged in this filed, aiming to explore the detailed molecular mechanism behind unsatisfactory survival outcomes. The potential positive effects of combining standard-of-care therapy with other novel drugs bring uplifting insight into applying a new therapeutic avenue for refractory prostate cancer [43]. For instance, transient receptor potential cation channel subfamily M member 8 (TRPM8) was proved to serve as a crucial role in advanced stage III/IV prostate cancers, and the TRPM8-induced calcium cytotoxicity could sensitize tumor cells to standard-of-care treatment, which provided additional clinical benefits [44]. The data from studies of mCSPC also indicated a correlation between the combination of ADT with chemotherapy [45,46] and radiotherapy or novel hormonal agents [47,48] and a better therapeutic response. However, the direct comparison on survival results including rPFS and OS between the combination of PARP inhibitors plus NHT and the sequential use of the two drugs (the current standard of care) has not been reported in all of the mentioned trials. The data of subsequent therapies (especially for patients randomly assigned to the NHT plus placebo arms who received PARP inhibitors in the latter stages) are not available. Even among the patients with BRCA2 m where the evidence is strongest, we still cannot conclude which therapy modality is the best to gain rPFS benefits. In the future, more prospective data of high quality are needed to confirm the combination-associated treatment efficacy and survival benefits based on the preclinical or clinical trials.

We conducted the current systematic review to summarize the evidence and present our findings narratively. However, because the majority of data about combining PARP inhibitors and NHT as a first-line therapy for mCRPC are still emerging, we did not critically evaluate the quality of our included trials by using quality assessment tools and perform any meta-analyses.

## 5. Conclusions

Based on the current data, combining AAP or ENZA with PARP inhibitors could achieve a significant survival benefit in the first-line mCRPC setting for patients with HRR m, especially with BRCA1/2 m. Furthermore, prior taxane-based chemotherapy might not influence the efficacy of the combination. It should be noted that all of the three trials lacked patients who received NHT in the mCSPC or nmCRPC setting before enrollment (3.1%, 0.3% and 5.2% in MAGNITUDE, PROpel and TALAPRO-2, respectively).

## Figures and Tables

**Table 1:** Published and on-going clinical trials of PARP inhibitors combining NHT for mCRPC in the first-line setting. AAP = abiraterone acetate plus prednisone; ENZA = enzalutamide; rPFS = radiographic progression-free survival; OS = overall survival; NHT = novel hormonal therapies; PARP = poly (ADP-ribose) polymerase; HRR = homologous recombination repair; HRR m = HRR mutation; BRCA1/2 m = BRCA1/2 mutation; n = number; NA = no data; AEs = adverse effects.

Clinical Trial	Treatment Arms		Patients	HRR Gene Panel	HRR m Status	Primary End Points	Other Reported End Points
**Trials reported data**							
MAGNITUDE (NCT03748641) [26,30,31]	Niraparib + AAP (n = 212 in HRR m cohort; n = 123 in non-HRR m cohort)	Placebo + AAP (n = 211 in HRR m cohort; n = 124 in non-HRR m cohort)	mCRPC, unselected patients, allowed ≦4 months first-line AAP (41 in non-HRR m cohort and 98 in HRR m cohort) in the mCRPC first-line setting; 3.1% (n = 31) and 20.1% (n = 85) included patients have NHT and taxane-based chemotherapy in mCSPC and/or nmCRPC stage in HRR m cohort, respectively.	Tissue and/or blood samples: ATM, BRCA1, BRCA2, BRIP1, CDK12, CHEK2, FANCA, HDAC2, PALB2	For HRR+ cohort: Niraparib + AAP: 46.3% (n = 98) BRCA1/2 m, 53.7% (n = 114) non-BRCA1/2 m; Placebo + AAP: 43.6% (n = 92) BRCA1/2 m, 56.4% (n = 119) non-BRCA1/2 m.	rPFS	Immature OS (at second interim analysis); AEs
PROpel (NCT03732820) [27,32]	Olaparib + AAP (n = 399)	Placebo + AAP (n = 397)	mCRPC, unselected patients, no prior systemic treatment for mCRPC; Only 1 patient received NHT at mCSPC stage; 22.6% and 22.4% patients received docetaxel at mCSPC stage in the combined arm and placebo arm, respectively.	Tissue and/or blood samples: ATM, BRCA1, BRCA2, BARD1, BRIP1, CDK12, CHEK1, CHEK2, FANCL, PALB2, RAD51B, RAD51C, RAD51D, RAD54L	Olaparib + AAP: 27.8% (n = 111) HRR m, 69.9% (n = 279) non-HRR m, 11.8% (n = 47) BRCA1/2 m; Placebo + AAP: 29.0% (n = 115) HRR m, 68.8% (n = 273) non-HRR m, 9.6% (n = 38) BRCA1/2 m.	rPFS	Final OS; AEs
TALAPRO-2 (NCT03395197) [28,33]	Talazoparib + ENZA (n = 402)	Placebo + ENZA (n = 403)	mCRPC, unselected patients, no prior systemic treatment for mCRPC; 5.2% (n = 21) and 6.2% (n = 25) patients received abiraterone at mCSPC stage in the combined arm and placebo arm; 21.4% (n = 86) and 23.1% (n = 93) patients received docetaxel at mCSPC stage in the combined arm and placebo arm.	Tissue and/or blood samples: BRCA1, BRCA2, PALB2, ATM, ATR, CHEK2, FANCA, RAD51C, NBN, MLH1, MRE11A, CDK12	Talazoparib + ENZA: 21.1% (n = 85) HRR m, 78.9% (n = 317) non-HRR m, 6.9% (n = 28) BRCA1/2 m; Placebo + ENZA: 20.3% (n = 82) HRR m, 79.7% (n = 321) non-HRR m, 7.9% (n = 32) BRCA1/2 m;	rPFS	Immature OS; AEs
**Trials not reported data**							
CASPAR (NCT04455750) [29,34]	Rucaparib + ENZA (n = 492)	Placebo + ENZA (n = 492)	mCRPC, unselected patients, no prior treatment for mCRPC; Prior NHT (except ENZA) and/or docetaxel chemotherapy at mCSPC and/or nmCRPC stage was allowed.	Tissue samples: NA	-	rPFS and OS	-

**Table 2:** Treatment-emergent adverse events. AAP = abiraterone acetate plus prednisone; ENZA = enzalutamide; n = number; AEs = adverse effects.

AEs, n (%)	MAGNITUDE (HRR m)				PROpel				TALAPRO-2			
	Niraparib + AAP		Placebo + AAP		Olaparib + AAP		Placebo + AAP		Talazoparib + ENZA		Placebo + ENZA	
	All Grades	Grade ≥ 3	All Grades	Grade ≥ 3	All Grades	Grade ≥ 3	All Grades	Grade ≥ 3	All Grades	Grade ≥ 3	All Grades	Grade ≥ 3
**Any AEs**	210 (99.1)	142 (66.9)	199 (94.3)	98 (46.5)	387 (97.2)	188 (47.2)	376 (94.9)	152 (38.4)	392 (98.0)	299 (75.0)	379 (95.0)	181 (45.0)
**Interruption due to adverse event**	-	-	-	-	178 (44.7)	-	100 (25.3)	-	300 (75.0)	-	94 (23.0)	-
**Dose reduction due to adverse event**	42 (19.8)	-	7 (3.3)	-	80 (20.1)	-	22 (5.6)	-	223 (56.0)	-	29 (7.0)	-
**Discontinuation due to adverse event**	23 (10.8)	-	10 (4.7)	-	55 (13.8)	-	31 (7.8)	-	76 (19.0)	-	49 (12.0)	-
**Blood and lymphatic system disorders**												
Anemia	98 (46.2)	63 (29.7)	43 (20.4)	16 (7.6)	183 (46.0)	60 (15.1)	65 (16.4)	13 (3.3)	262 (66.0)	185 (46.0)	70 (17.0)	17 (4.0)
Thrombocytopenia	45 (21.2)	14 (6.6)	18 (8.5)	5 (2.4)	-	-	-	-	98 (25.0)	29 (7.0)	14 (3.0)	4 (1.0)
Neutropenia	29 (13.7)	14 (6.6)	12 (5.7)	3 (1.4)	-	-	-	-	142 (36.0)	73 (18.0)	28 (7.0)	6 (1.0)
Leukopenia	22 (10.4)	4 (1.9)	5 (2.4)	1 (0.5)	-	-	-	-	88 (22.0)	25 (6.0)	18 (4.0)	0 (0)
**Cardiac disorders**												
Hypertension	66 (31.1)	31 (14.6)	44 (20.9)	26 (12.3)	50 (12.6)	14 (3.5)	65 (16.4)	13 (3.3)	55 (14.0)	21 (5.0)	62 (15.0)	30(7.0)
Arrhythmia	27 (12.7)	6 (2.8)	-	-	-	-	-	-	-	-	-	-
**General disorders**												
Fatigue	56 (26.4)	7 (3.3)	35 (16.6)	9 (4.3)	148 (37.2)	9 (2.3)	112 (28.3)	6 (1.5)	134 (34.0)	16 (4.0)	118 (29.0)	8 (2.0)
**Gastrointestinal disorders**												
Constipation	65 (30.7)	0 (0)	29 (13.7)	0 (0)	69 (17.3)	0 (0)	55 (13.9)	1 (0.3)	72 (18.0)	1 (<1.0)	68 (17.0)	2 (<1.0)
Nausea	50 (23.6)	1 (0.5)	29 (13.7)	0 (0)	112 (28.1)	1 (0.3)	50 (12.6)	1 (0.3)	82 (21.0)	2 (<1.0)	50 (12.0)	3 (<1.0)
Diarrhea	-	-	-	-	69 (17.3)	3 (0.8)	37 (9.3)	1 (0.3)	57 (14.0)	1 (<1.0)	55 (14.0)	0 (0)
Decreased appetite	30 (14.2)	1 (0.5)	13 (6.2)	1 (0.5)	58 (14.6)	4 (1.0)	23 (5.8)	0 (0)	86 (22.0)	5 (1.0)	63 (16.0)	4 (1.0)
**Hepatotoxicity**	25 (11.8)	4 (1.9)	-	-	-	-	-	-	-	-	-	-
**Back pain**	31 (14.6)	5 (2.4)	44 (20.9)	2 (0.9)	67 (17.1)	3 (0.8)	73 (18.4)	4 (1.0)	88 (22.0)	10 (3.0)	72 (18.0)	4 (1.0)
**Arthralgia**	28 (13.2)	2 (1.0)	20 (9.5)	1 (0.5)	51 (12.8)	0 (0)	70 (17.7)	2 (0.5)	58 (15.0)	2 (<1.0)	79 (20.0)	2 (<1.0)
**Urinary tract infection**	-	-	-	-	41 (10.3)	8 (2.0)	31 (7.8)	4 (1.0)	-	-	-	-

## Data Availability

All data used in this research can be found in the article and references.

## Supplementary Materials

The following supporting information can be downloaded at: https://www.mdpi.com/article/10.3390/curroncol30120751/s1,Table S1: Information of Included records; Table S2: Sensitive analyses of the MAGNITUDE trial for patients received AAP in the mCRPC setting.
