# Supplementary material for: Combining Novel Hormonal Therapies with a Poly (ADP-Ribose) Polymerase Inhibitor for Metastatic Castration-Resistant Prostate Cancer: Emerging Evidence
Source: Curr Oncol. 2023 Dec 4;30(12):10311–24. doi: 10.3390/curroncol30120751 (PMC10742907; doi:10.3390/curroncol30120751)
Supplement: Supplementary file 1 [file curroncol-30-00751-s001.zip › Supplemental Table S1.pdf]

Supplemental Table S1. Information of Included records.

Reference	NCT Number	Acronym
**Publications**		
Chi KN et al. J Clin Oncol. 2023;O2201649	NCT03748641	MAGNITUDE
Clarke NW et al. NEJM Evid. 2022;1:10.1056/EVIDoa2200043	NCT03732820	PROpel
Agarwal N et al. Lancet. 2023;402:291-303	NCT03395197	TALAPRO-2
**ASCO-GU abstracts**		
Castro E et al. J Clin Oncol. 2023.41.6_suppl.172	NCT03748641	MAGNITUDE
Efstathiou E et al. J Clin Oncol. 2023.41.6_suppl.170	NCT03748641	MAGNITUDE
Clarke NW et al. J Clin Oncol. 2023.41.6_suppl.LBA16	NCT03732820	PROpel
Agarwal N et al. J Clin Oncol. 2023.41.6_suppl.LBA17	NCT03395197	TALAPRO-2
Rao A et al. J Clin Oncol. 2023.41.6_suppl.TPS277	NCT04455750	CASPAR
**On-going Clinical Trials**		
https://clinicaltrials.gov/ct2/show/NCT04455750	NCT04455750	CASPAR
