# Supplementary material for: Combining Novel Hormonal Therapies with a Poly (ADP-Ribose) Polymerase Inhibitor for Metastatic Castration-Resistant Prostate Cancer: Emerging Evidence
Source: Curr Oncol. 2023 Dec 4;30(12):10311–24. doi: 10.3390/curroncol30120751 (PMC10742907; doi:10.3390/curroncol30120751)
Supplement: Supplementary file 1 [file curroncol-30-00751-s001.zip › Supplemental Table S2.pdf]

Supplemental Table S2. Sensitive analyses of the MAGNITUDE trial for patients received AAP in the mCRPC setting. AAP=Abiraterone Acetate plus Prednisone; HR=Hazard Ratio; N=Number; CI=Confidence Interval; HRR=Homologous Recombination Repair; HRR m=HRR mutation; BRCA1/2 m=BRCA1/2 mutation.

**Cohort**	**Subgroup**	**N**	**HR**	**95%CI**	**Median rPFS, months**	
					**Niraparib+AAP**	**Placebo+AAP**
HRR m	No prior AAP	325	0.72	(0.55, 0.96)	19.4	11.2
	AAP ≦ 2 months	58	0.69	(0.36, 1.30)	13.9	11.1
	AAP > 2 months	40	1.47	(0,66, 3.30)	13.1	16.5
BRCA1/2 m	No prior AAP	166	0.48	(0.32, 0.71)	19.6	8.4
	AAP ≦ 2 months	36	0.67	(0.27, 1.47)	13.9	11.1
	AAP > 2 months	23	1.20	(0.30, 4.80)	NR	24.9
